# Electroacupuncture attenuates LPS-induced depression-like behavior through kynurenine pathway

**DOI:** 10.3389/fnbeh.2022.1052032

**Published:** 2023-01-10

**Authors:** Xingying Wu, Rong Hu, Shuo Jiang, Zhong Di, Yi Chen, Mengting Shi, Bowen Chen, Kelin He, Kecheng Qian, Qin Guo, Ruijie Ma

**Affiliations:** ^1^Key Laboratory of Acupuncture and Neurology of Zhejiang Province, Department of Neurobiology and Acupuncture Research, The Third School of Clinical Medicine (School of Rehabilitation Medicine), Zhejiang Chinese Medical University, Hangzhou, China; ^2^Department of Acupuncture and Moxibustion, The First Affiliated Hospital of Zhejiang Chinese Medical University, Hangzhou, China; ^3^Department of Acupuncture and Moxibustion, The Third Affiliated Hospital of Zhejiang Chinese Medical University, Hangzhou, China

**Keywords:** electroacupuncture, depression, LPS, kynurenine pathway, IDO

## Abstract

**Background:**

A growing body of evidence suggests that inflammation and changes in glutamate neurotransmission are two pathophysiological mechanisms underlying depression. Electroacupuncture (EA) is a common therapeutic tool for the treatment of depression. However, the potential antidepressant mechanism of EA remains obscure. The change of the kynurenine pathway (KP) is the research priority of antidepressant mechanisms. This study will investigate the role of EA on lipopolysaccharide (LPS)-induced depression-like behavior and explore its possible mechanism of action.

**Methods:**

Lipopolysaccharide was used to induce depression-like behavior, and EA was given at Hegu (L14) and Taichong (LR3) acupoints in C57BL/6J mice. Depression-like behaviors were measured by behavioral tests, including tail suspension test (TST), sucrose preference test (SPT), force swim test (FST), and open field test (OFT). The levels of inflammatory cytokines IL-1β, IL-6, and TNF-α, and KP enzyme IDO1 were measured by qPCR and enzyme-linked immunosorbent assay (ELISA), while high-performance liquid chromatography (HPLC) was performed to detect the content of prefrontal cortex and hippocampal as well as serum glutamate, tryptophan (TRP), kynurenic (KYN), and quinolinic acid (QA).

**Results:**

The results showed that (1) as evidenced by increased spontaneous locomotor activities, decreased immobility duration, and a stronger preference for sucrose in the sucrose preference test, EA reversed LPS-challenged depressive-like behavior. (2) EA at L14 and LR3 decreased the levels of inflammatory cytokines, inhibited IDO1, and regulated KP metabolisms, as well as lowered the concentration of glutamate. (3) EA may exert anti-depression effects by acting on the kynurenine pathway.

**Conclusion:**

This study evaluated the effects of EA on depression-like behaviors induced by lipopolysaccharide (LPS) and its regulation of inflammation and the glutamatergic system. Our results suggest that EA can ameliorate depression-like behaviors, lower the level of inflammation, and reduce the release of glutamate, possibly through the regulation of the kynurenine pathway in the brain.

## Introduction

Depression affects 16% of the world’s population and has serious health and socioeconomic consequences. Increasing evidence suggests that depression is involved in oxidative stress and pro-inflammatory cytokines. In particular, widespread attention has been paid to the role inflammation plays in depression, which is caused by neurodegenerative metabolites in the CNS (Kohler et al., 2016). Furthermore, the inflammation affects glutamate release, transmission, and metabolism, leading to excessive extracellular concentrations of glutamate in the brain, further worsening depression. One potential intersection point of these three is the kynurenine (KYN) pathway (Tao et al., 2020).

A peripherally injected inflammatory stimulus, for instance, lipopolysaccharide (LPS) or Bacillus Calmette–Guérin (BCG) vaccine, induces the kynurenine pathway (KP) by activating the enzyme indoleamine 2,3-dioxygenase (IDO), which is involved in the onset of KP and converts tryptophan (TRP) to kynurenine (KYN) (Dantzer, 2017; Arnone et al., 2018), and thus produced further various metabolites including quinolinic acid (QA), an endogenous convulsant compound that overstimulates the glutamatergic system by stimulating N-methyl-D-aspartate receptors (NMDAR), thereby inhibiting glutamate uptake and enhancing glutamate release (Schetinger et al., 2007). Interestingly, in mice, the NMDAR antagonist ketamine significantly reduced LPS-induced depressive behaviors, which are also mediated by IDO1, without affecting inflammation cytokines (Walker et al., 2013). Overexpressed cytokines interfere with kynurenine binding, glutamate synthesis, and glutamate release during inflammation, in the limbic system, resulting in a higher level of extracellular glutamate (Haroon and Miller, 2017; Cui et al., 2019).

The development of several IDO inhibitors is currently underway (for treating cancer) and might be used to treat depressed patients with inflammatory activation of the KP (Haroon et al., 2020). Moreover, KP metabolites’ downstream effects on glutamate neurotransmission may be modulated by glutamate receptor modulators, such as ketamine, memantine, and riluzole (Haroon and Miller, 2017). However, the current therapeutic drugs have shortcomings such as long onset time and short effect time. Thus, there is a need to develop an effective treatment for depression that has no side effects and is low-cost. To stimulate acupuncture points, electroacupuncture (EA) uses a mild electrical current in conjunction with traditional acupuncture, which offers numerous advantages over traditional antidepressant options (First et al., 2011; Yang et al., 2013; Xie et al., 2016), such as soothing the liver and regulating qi in addition to soothing the mind and relieving depression, and is better accepted and tolerated with fewer side effects.

However, whether electroacupuncture treats depression through KP is unknown. Therefore, we established an inflammation-induced depression model and measured depression-like behaviors, inflammatory cytokines, glutamate level, and kynurenine metabolisms to find to measure the function of EA on depression. We observed EA could treat pathological inflammation (Zhang et al., 2015), and as observed in the tail suspension test (TST) and forced swimming test (FST) behavioral responses, EA significantly attenuated depressive behavior (Lottering and Lin, 2021). Moreover, in the nervous system, EA could ameliorate glutamate and regulate kynurenine metabolisms.

## Materials and methods

### Animals and LPS-induced model

C57BL/6J male mice (8–10 weeks old) (Laboratory Animal Center of Zhejiang Chinese Medicine University) with approval number 20210315-15 were used in these experiments. We gave the mice a minimum of 1 week to acclimatize to the new environment before conducting the experiment and were raised five per cage access to food and water *ad libitum* in a standard environment (25 ± 2°C and 12:12-h light–dark cycle). Experiments on animals were conducted in a separate procedure room outside of their housing area. The study period was characterized by the use of ventilation and air filtration systems. During the experiment, laboratory animals were treated according to the Guidance Opinions on the Ethical Treatment of Laboratory Animals issued in 2006 by the Ministry of Science and Technology, PRC.

Lipopolysaccharide (L-3129, serotype 0127: B8; Sigma) was diluted by a sterile endotoxin-free phosphate-buffered saline (PBS) solution. Mice received 0.83 mg/kg LPS or vehicle (equivolume) for studies of depression induced by inflammation (Walker et al., 2019). We selected this dose of LPS because it reliably induced acute sickness behavior and depression-like behaviors across the time points studied (O’connor et al., 2009).

### Drugs

The IDO1 inhibitor, 1-methyl-L-tryptophan (1-MT, Sigma-Aldrich, USA), was prepared in an intraperitoneal dose of 15 mg/kg/d for 3 days (48 h, 24 h, and 30 min prior to LPS) (Salazar et al., 2012). Based on the solubility of this inhibitor, the drug powder was dissolved in 0.1 M sodium hydroxide and the pH was adjusted to 9.0 using hydrochloric acid with a 1:1 dilution volume ratio before administration (Deng et al., 2021).

### Electroacupuncture treatment

We randomly divided the mice into five groups (*n* = 8): (a) the PBS group, (b) the LPS group, (c) the 1-MT group, (d) the electroacupuncture group (EA), and (e) the sham electroacupuncture group (Sham EA), and the PBS group was intraperitoneally injected with physiological saline (5 ml/kg). Other groups received intraperitoneal injections of LPS (0.83 mg/kg). In the EA group, mice were immobilized by adhesive tape and stimulated on Hegu (L14) and Taichong (LR3) for 45 min by sterilized stainless steel needles (HANS-200E; Huawei Co., Ltd., Beijing, China), and the diameter and length were 0.16 and 7 mm, respectively. The EA parameters were set as follows: frequency: 15 Hz, intensity: 0.3 mA, and time: 45 min. Electroacupuncture was intervened for 2 h before LPS injection, and 5 and 23.5 h after LPS injection, and Sham EA treatment consisted of needles inserted into L14 and LR3, but no electrical stimulation was applied.

### Behavioral tests

#### Bodyweight measurement

To investigate alterations in mice’s physical conditions during the LPS-challenged, an electronic balance was used to determine the weight of each mouse 2, 6, and 24 h after LPS (O’connor et al., 2009).

#### Sucrose preference test

All mice were presented with 1% sucrose for 24 h, and the bottle size was consistent. The mice were given identical bottles of 1% sucrose and water after being dehydrated for 6 h. Three hours later, the bottles were removed and weighed to determine the fluid consumption.

#### Open field test

To discard unspecific effects, all experimental mice were subjected to the OFT. In a black rectangular box with a square floor (45 cm × 45 cm × 45 cm), nine equal-sized zones (15 cm × 15 cm) were created. A video tracking system (Ethovision, Nodus Information Technology) was used to videotape the behavior of mice in the central zone in an open field arena over a period of 5 min.

#### Forced swim test

**FIGURE 1:**
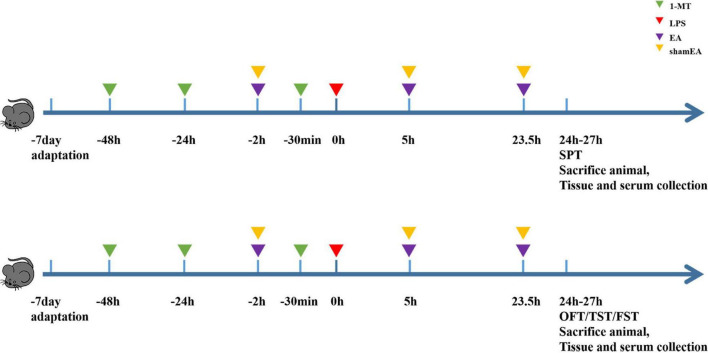
Experimental design scheme. Mice were acclimated for 7 days and received the administration of 1-MT (15 mg/kg, i.p.) before 48 h, 24 h, and 30 min of administrating LPS (0.83 mg/kg, i.p.); electroacupuncture and sham electroacupuncture treatment were performed once before LPS injection and repeated 5 and 23.5 h after LPS injection; behavior tests were done at 24–27 h after LPS. Each group contains four behavior tests as follows: sugar preference, open fields, forced swimming, and suspension of the tail.

Each mouse was placed into a cylinder made of glass (height: 21 cm and diameter: 14.5 cm), with 15 cm of water at a temperature of 23 + 1°C. They were forced to swim for 6 min (test) and exhibit immobility behavior when mice remain afloat and tread just enough to keep their noses above water, and then their total immobility time was measured. Swimming sessions were followed by careful drying in heated cages, followed by the return of the mice to their cages after 20 min. All experimental sessions were videotaped and later analyzed. The experimental design scheme is shown in Figure 1.

### Biochemical analysis

#### Measurements of IDO1 in the brain tissue and serum

The brain and serum were collected to assay IDO1 levels. The serum was separated from naturally clotted blood and then centrifuged at 3,000 rpm for 10 min. PBS (1 ml/10 mg) in an appropriate volume was added into a portion of the brain samples. After homogenization and centrifugation, samples were centrifuged for 20 min (3,000 rpm) at 4°C, and for further analysis, the supernatant was collected. Based on the protocols provided with the ELISA kits (JL47052) (Jianglai, Shanghai, China), we determined the IDO1 levels. Each sample was measured at 450-nm wavelength.

#### Measurement of cytokines

It collected serum and brain tissues from mice for cytokine determination. Cytokine levels were measured according to the instructions provided with ELISA kits for IL-6 (JL20268), IL-1 beta (JL JL18442), and TNF-α (JL10484) (Jianglai, Shanghai, China). At 450 nm, each sample’s absorbance (OD value) was measured.

#### Measurement of Glu and KP metabolism in the brain tissue and serum

A Glu and KP metabolism assay was performed on the brain tissue and serum of the animals. All metabolisms were using an HPLC-1100 system (Agilent Technologies, USA) equipped with a quaternary pump, and Glu, TRP, and KYN were measured by a UV detector, while QA was fluorescence. The samples were analyzed with an Agilent C18 column (5 micron particle size, L × I.D. 25 cm × 4.6 mm) preceded by a C18 guard column (Agilent Technologies, USA). For TRP and KYN, in the mobile phase, 6% acetonitrile was dissolved in a buffer containing 15 mM acetic acid–sodium acetate (pH 5.3) (Liang et al., 2019). For Glu, the gradient elution (GQ) system consisted of 0.05 mol/L sodium acetate buffer (pH = 6) and acetonitrile/water (V/V = 50:50). For QA, as mobile phase solvents, acetonitrile formate (0.1%) and formic acid (0.1% v/v) were added to water and methanol (Gibney et al., 2014).

### Quantitative real-time PCR analysis

Based on the manufacturer’s instructions, total RNA was extracted from the brain hippocampus (HIP) and prefrontal cortex (PFC) of mice killed by rapid decapitation using TRIzol reagent (Invitrogen, Carlsbad, USA) and synthesized cDNA using the Prime Script First-Strand cDNA Synthesis Kit (Takara Biotechnology). The following specific primers are used in Table 1.

**TABLE 1:** Sequences of the primers used for qPCR.

Sequence name	Primers′ sequence (5′ to 3′)	Amplicon size (bp)
Actin	F: CACCCGCGAGTACAACCTTC R: CCCATACCCACCATCACACC	207
IL-6	F: AGAGACTTCCAGCCAGTTGC R: CTGGTCTGTTGTGGGTGGTA	115
IL-1β	F:CAACTGTTCCTGAACTCAACTG R:GAAGGAAAAGAAGGTGCTCATG	290
TNF-α	F: GATCGGTCCCAACAAGGAGG R: GCTTGGTGGTTTGCTACGAC	138
IDO1	F: CGAGAACATGGACATTCTGTTC R: TTTCCAATGCTTTCAGGTCTTG	316

### Statistical analysis

GraphPad Prism 8 (GraphPad Software, San Diego, CA, USA) was used for statistical analysis. Mean ± SEM was expressed for the results. Student’s *t*-test was used for comparisons between the two groups. ANOVA followed by Tukey’s *post hoc* test was used for comparisons among groups. When *p* < 0.05, the comparison is considered significant.

## Results

### LPS-induced depression-like behavior

**FIGURE 2:**
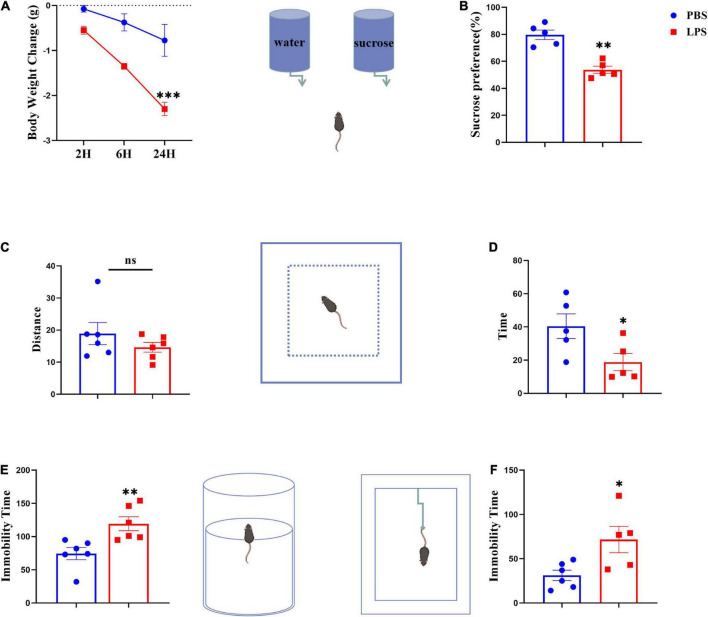
Lipopolysaccharide-stressed mice exhibit depression-like behaviors. The body weight changes and sucrose preferences in LPS-stressed mice are shown in panels **(A,B)**. Locomotor activity was evaluated as shown in panel **(C)**. The depression-like behaviors were tested by the time spent in central zones in OFT **(D)**, and immobility time in FST **(E)** and TST **(F)**. Data were expressed as the mean ± SEM (*n* = 5 per group). *p < 0.05, **p < 0.01, ***p < 0.001, compared with the saline group. *ns*, no statistical significance.

Animals were treated with LPS (0.83 mg kg^–1^) or saline with a single i.p. injection for the purpose of inducing sickness or depression-like behavior. Before being euthanized, mice were weighed and tested, and then collected the serum and brain samples. We assessed changes in body weight (*p* < 0.001) (Figure 2A) and the sucrose preference test (*p* < 0.01) (Figure 2B) at 24 h after LPS injection to determine whether sickness was induced. The total distances traveled by mice during the 5-min OFT test were used to evaluate their locomotor activity. There were no significant differences between all mice tested, indicating LPS had no effect on locomotor activity (Figure 2C). As expected, compared with controls, LPS mice spent significantly less time exploring the central areas, suggesting that inflammatory events may decrease exploratory behavior (*p* < 0.05) (Figure 2D). Furthermore, LPS-challenged mice prolonged immobility time in both forced swim (*p* < 0.01) and tail suspension tests (*p* < 0.05) (Figures 2E, F), and there have been reports of despair-like behavior in stressed mice, which has been interpreted as a sign of depression by others (Zhao et al., 2019).

### Electroacupuncture blocks LPS-induced cytokine and IDO expression

**FIGURE 3:**
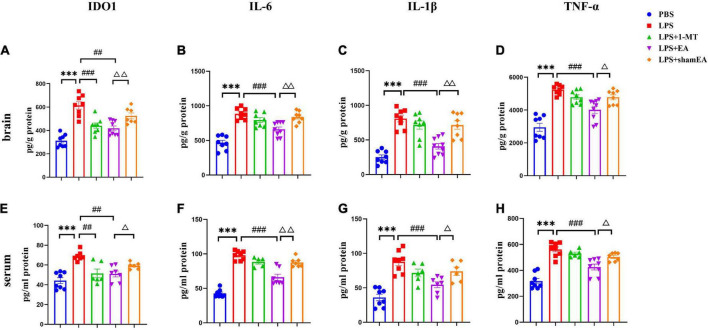
Electroacupuncture reduced the level of IDO1, IL-6, IL-1β, and TNFα in the brain tissue and serum, which were induced by LPS of mice. After being killed, the tissue and serum of mice were collected and analyzed for protein level in the peripheral **(A–D)** and center **(E–H)** of **(A,E)** IDO1, **(B,F)** IL-6, **(C,G)** IL-1β, and **(D,H)** TNFα using ELISA. The results are expressed as the mean ± SEM (*n* = 8 per group. ***p < 0.001, compared with the saline group; *^##^p* < 0.01, *^###^p* < 0.001, compared with the LPS group; ^Δ^*p* < 0.05, ^ΔΔ^*p* < 0.01, compared with the sham EA group).

**FIGURE 4:**
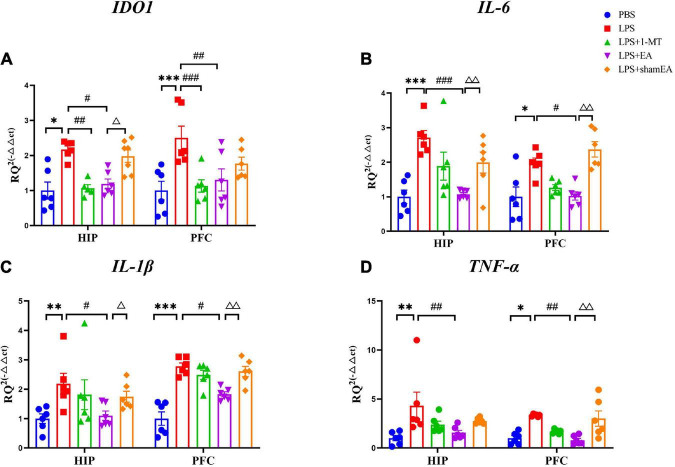
Effects of EA have been observed in the hippocampi and prefrontal cortex of mice, which were upregulated by LPS. Mouse HIP and PFC were collected after behavior testing and tested for mRNA levels of **(A)** IDO1, **(B)** IL-6, **(C)** IL-1β, and **(D)** TNF α using quantitative PCR. The results are expressed as the mean ± SEM (*n* = 8 per group. *p < 0.05, **p < 0.01, ***p < 0.001, compared with the saline group; *^#^p* < 0.05, *^##^p* < 0.01, *^###^p* < 0.001, compared with the LPS group; ^Δ^*p* < 0.05, ^ΔΔ^*p* < 0.01, compared with the sham EA group).

To confirm the EA had an effect on inflammation, brain and serum samples were collected 27 h after the administration of LPS. As shown in Figure 3, the protein expression levels of IL-6, IL-1β, and TNFα in the brain and serum were greater than those in the saline group and were significantly increased after injected with LPS (*p* < 0.0001) (Figures 3B–D, F–H, respectively), all of which were inhibited by electroacupuncture intervention (*P* < 0.01) than that in the model group. The 1-MT treatment produced no obvious decrease of inflammatory cytokines in the brain and serum compared with the model group. Compared with the EA group (*p* < 0.05), the sham EA group did not show any difference in pro-inflammatory cytokine levels. PCR results were consistent with those obtained using ELISA. The qPCR results showed that LPS significantly increased IL-6, Il-1β, and TNF-α mRNA expression in brain tissues, increases which were significantly attenuated by EA (Figures 4B–D). Similar to LPS, sham EA and 1-MT had no significant effect on overexpression of IL-6, Il-1β, and TNF-α mRNA expression (Figures 4B–D).

We also tested the protein and gene levels of IDO1, which is activated by inflammatory factors or stress hormones (Wang et al., 2017). Compared with the control group, the protein and gene levels of IDO1 showed a remarkable increase in the model groups, while after 1-MT or EA interventions, the levels of IDO1 decreased significantly in both groups (*p* < 0.05, Figures 3A, E, 4A), and the sham EA group did not show any difference in IDO1 levels compared with the EA group. From the aforementioned results, some interesting conclusions can be drawn, and electroacupuncture, such as 1-MT, can significantly inhibit IDO1, indicating that electroacupuncture may regulate KP by intervening IDO. The difference is that electroacupuncture has an obvious anti-inflammatory effect but 1-MT does not, indicating that electroacupuncture may reduce the expression of IDO by reducing inflammation.

### Electroacupuncture inhibits IDO by the kynurenine pathway

**FIGURE 5:**
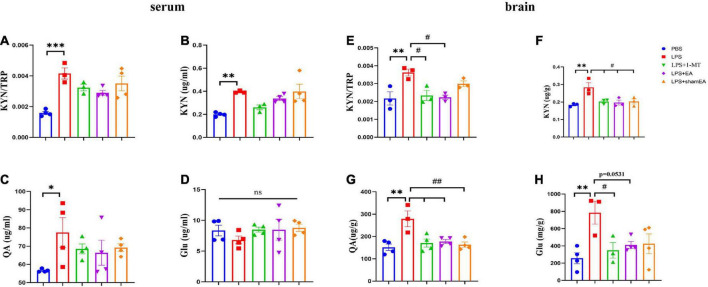
Electroacupuncture regulates concentration levels of TRP: KYN, KYN, QA, and Glu. After behavior tests, the tissue and serum of mice were collected and analyzed for the metabolism level of KYN **(B,F)**, QA **(C,G)**, and QA **(D,H)**, and the ratio of KYN to TRP **(A,E)**. The results are expressed as the mean ± SEM. *p < 0.05, **p < 0.01, ***p < 0.001, compared with the saline group; *^#^p* < 0.05, *^##^p* < 0.01, compared with the LPS group; *ns*, no statistical significance.

To explore whether EA regulates kynurenine pathway, we assessed the concentrations of TRP, KYN, QA, and Glu in peripheral and central. All of the peripheral and central TRP levels varied little throughout the whole experiment (data not shown). Consistent with the existing literature, we found that serum kynurenine and quinolinic acid increased in LPS-treated mice (Figures 5B, C) (*p* < 0.05); meanwhile, an increase in the ratio of serum kynurenine to tryptophan had been found (Figure 5A) (*p* < 0.001). The KYN/TRP ratio was to express the IDO-1 activity (Sundaram et al., 2020). Glutamate, an excitatory neurotransmitter in the central nervous system, could initiate depression (Haroon et al., 2017). However, it was not greatly changed in the serum (Figure 5D). There was a significant increase in the brain kynurenine/tryptophan ratio (*p* < 0.01) (Figure 5E), which was attenuated by 1-MT and EA intervention (*p* < 0.05) (Figure 5E). Correspondingly, increases in the concentrations of KYN, QA, and Glu were also observed (Figures 5F–H) (*p* < 0.01), which were significantly decreased by both 1-MT and EA (Figure 5H, *p* = 0.0531). To our surprise, sham EA shows a similar inhibitor effect on the KYN and QA (Figures 5F, G). We speculate that sham EA may act through other mechanisms, which need further research. These results indicated that systemic inflammatory response could regulate the tryptophan-kynurenine pathway, and EA may play an antidepressant effect role by regulating KP.

### Electroacupuncture relieves LPS-induced depression-like behavior

**FIGURE 6:**
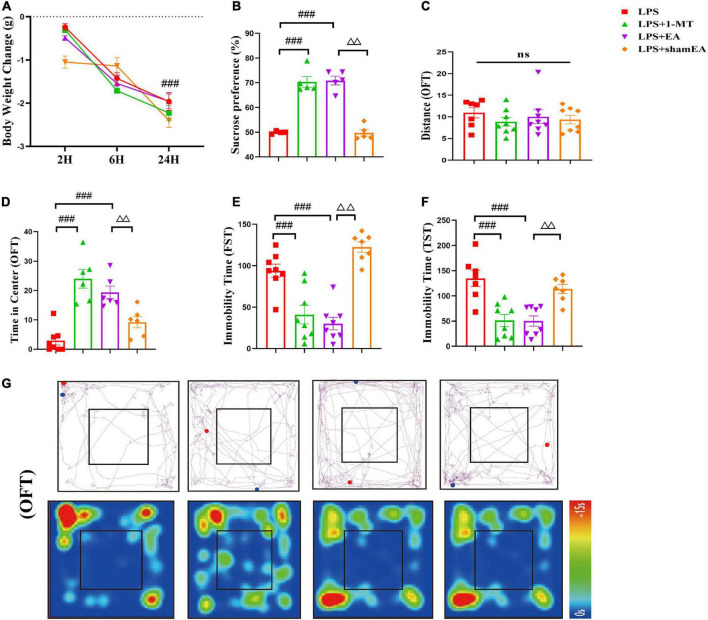
Electroacupuncture alleviates depression-like behavior induced by LPS in mice. Illness severity indicators were assessed, including **(A)** weight loss, **(B)** sucrose preference test, and **(C)** total distance traveled in the open field test (OFT). Depression-like behavior was evaluated by **(D)** time spent in the center square and effects of EA on LPS-induced locomotor activity and cognitive functions in mice **(G)**, immobility time in FST **(E)**, and immobility time in TST **(F)**. All data were expressed as the mean ± SEM (*n* = 8 per group). ^###^*p* < 0.001, compared with the LPS group; ^ΔΔ^*p* < 0.01, compared with the sham EA group. *ns*, no statistical significance.

After we investigated EA’s effects on the kynurenine pathway, we observed the function of EA on depression-like behaviors. As indicated in Figure 6A, the EA group did not show any difference in the change in body weight 24 h compared with the model group, and 1-MT and sham EA had no effect on reduction in body weight in response to LPS (Figure 6A). In the forced swim test and tail suspension test, LPS increased the duration of immobility and was completely ablated in 1-MT or EA-treated mice (*p* < 0.001) (Figures 6E, F). Similarly, a marked decrease in the time in the center was inhibited by the treatment with 1-MT and EA (*p* < 0.001) (Figure 6D). Moreover, the sucrose preference test decreased significantly in response to LPS, but this decrease was rescued in the 1-MT group and EA group (*p* < 0.001) (Figure 6B). Moreover, the effect of improving the depression-like behavior was greater in the EA group (*p* < 0.01) than in the sham EA group. As we expected, no changes in total horizontal locomotion distance were observed among these groups (Figure 6C). These data establish EA as a novel therapeutic tool in the treatment of depression-like behaviors induced by peripheral inflammation.

## Discussion

Growing evidence has shown that depression is widespread and has a great negative impact on not only psychological health but also jeopardizes the body. Thus, it is crucial to gain an effective therapeutic treatment. In recent years, the mechanism of treatment for depression was sought by a growing number of studies, and main several possible mechanisms including (Kou et al., 2017) (1) controlling the level of neurotransmitters, such as monoamines, glutamate, and GABA (Luo et al., 1998); (2) regulating neuroendocrine system [MT, HPT axis, and HPA axis (Xu et al., 2016)]; and (3) regulating inflammatory cytokines, such as IL-6, IL-1β, and TNF-α (Lu et al., 2017); while inflammation-induced depression is common and inflammation and depression are thought to be linked by KP metabolites through their effects on brain glutamate receptors (Haroon et al., 2020).

At present, some IDO inhibits are developing, and symptoms of depression can be treated clinically with western medicine approaches, but these approaches have side effects, including anxiety symptoms, sexual dysfunction, and gastrointestinal reactions (Han et al., 2021). Thus, it is necessary to explore new treatment strategies. Generally, electroacupuncture (EA) is considered a safe and harmless method of treating depression. Multiple therapeutic mechanisms are involved in the treatment of depression by EA, including neurotransmitters and neuropeptides are regulated, neural activity is modulated, and HPA axis hyperactivity, as well as inflammation, is inhibited. However, whether the EA modulates KP by inhibiting IDO1 improves depression-like behavior is unclear.

We explored that the antidepressant effect of EA on LPS-induced depression model may be through the KP, which is also the innovation of this paper. In the present study, we used a validated model of inflammation-induced depression along with four different behavioral measures of depression-like behavior to show that in response to the LPS challenge, the peripheral immune response was elicited, which triggered central inflammation, resulting in kynurenine pathway activation together with depression-like behaviors.

In our experiment, we chose the acupoint Hegu (LI 4), which is located at the midpoint of the second metacarpal on the radial side, and the acupoint Taichong (LR 3), which is located at the end of the second toe tibial collateral, in the rear of the phalanx (Xie et al., 2016). Because we previously showed that Hegu and Taichong acupoints could ameliorate the chronic unpredictable mild stress (CUMS) induced depression-like behavior (Guo et al., 2021), in particular, the network involved in emotion cognition within the functional brain was activated (Ji et al., 2021). We set up the Sham EA group as controls and 1-MT, a classical IDO1 inhibitor, as a positive control for the LPS-to-depression pathway is mediated by the activation of the ubiquitous enzyme IDO and increased the production of excitotoxic kynurenine metabolites, including quinolinic acid (QA), in the brain (Shi et al., 2019), thereby directly or indirectly affecting glutamate (Connick and Stone, 1988), leading to neuroexcitatory and neurotoxic properties.

As a result, the effect of EA was similar to pharmacologic blockade of IDO1, 1-MT, abrogating the IDO activity and decreasing the content of KYN and QA to protect from inflammation-induced IDO-dependent neurotoxic kynurenine metabolism, which was a pathogenic factor for inflammation-induced depressive disorders and a potential novel target for the treatment of these disorders (Heisler and O’connor, 2015). QA is a powerful and potent excitotoxin, which is sufficient to cause excitotoxicity and release excessive glutamate under pathological conditions (Haroon et al., 2017). In our result (Figure 6), EA decreased the concentration of QA and glutamate in the brain, suggesting it can exert neuroprotection in animal experiments. Nonetheless, the effect of EA on the peripheral kynurenine pathway was not obvious, which needs further examination.

To confirm that EA was affecting central or peripheral inflammation and depressive behavior in our model, we examined behavioral tests and inflammatory responses. Figures 3, 4, 6 demonstrated that depression-like behavior and inflammatory markers were altered greatly by EA. However, it was not changed by 1-MT or sham EA group. Therefore, it is highly possible that the beneficial actions of EA are partly and significantly dependent on the alleviation of peripheral and central inflammatory responses as well as glutamate by regulating KP.

As inflammation-induced depression is characterized by acute inflammatory stimulus and lack of chronicity characteristic of major depression, whether EA can eliminate depression-like behavior associated with chronic inflammatory conditions, or ensure no side effects, may be determined and needed further research. A potential regulatory effect of EA on other brain regions is also worth considering, since a systemic LPS challenge causes glial activation and metabolic changes in several brain regions, including the hippocampus, prefrontal cortex, and striatum; together, these factors shape neuroinflammation and neuronal function. Finally, we find sham EA had effects on KYN and QA in the brain, which was somewhat unexpected for us. However, there is another rate-limiting enzyme, TDO, to regulate circulate kynurenine, and then transported into the brain by the large amino transporter LAT1 through the blood–brain barrier to induce the activation of the brain kynurenine pathway, and sham EA may act through changes in those conditions; however, this needs to be further examined in future research. In the current study, we found that EA may play a key antidepressant and modulate inflammatory and glutamate roles by acting on KP.

## Conclusion

**FIGURE 7:**
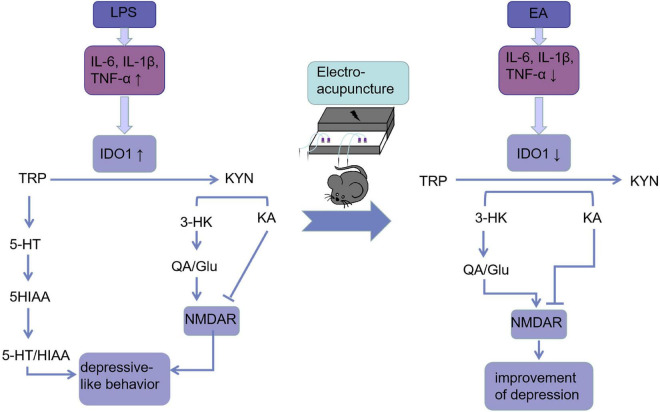
Electroacupuncture linking IDO-mediated TRP mechanism pathway to behavioral changes: EA decreased circulating and cerebral pro-inflammatory cytokines after the LPS challenge, which deactivate IDO1. When IDO1 is activated, most of the TRP is metabolized into KYN, which is converted into KYNA under normal conditions. However, when IDO1 is activated, most of KYN is converted to QA, an NMDA receptor agonist that is a key factor in increasing neurotoxicity and cognitive deficits. EA ameliorated these imbalances and behavioral deficits may be through inhibiting IDO1.

As a conclusion, we showed that EA can be used as an alternative therapy for treating mice with depression-like behaviors. A model of acute inflammation-induced depression is presented in Figure 7 along with our findings concerning EA interference with that pathway.

## Data availability statement

The original contributions presented in this study are included in the article/supplementary material, further inquiries can be directed to the corresponding authors.

## Ethics statement

The animal study was reviewed and approved by the Laboratory Animal Center of Zhejiang Chinese Medicine University.

## Author contributions

XW contributed to this work. RM and QG designed this study. XW, RH, SJ, and ZD analyzed the data. XW, YC, and MS wrote the manuscript. BC, KH, and KQ participated in the revision of the article. RM and QG validated the manuscript. All authors had read and approved the final version of the manuscript.
